# Xanthogranulomatous pyelonephritis presenting as a cystic mass: a rare case report

**DOI:** 10.1093/jscr/rjab265

**Published:** 2021-06-22

**Authors:** Mohamed Hafedh Saadi, Mokhtar Bibi, Issam Rekik, Yassine Ouanes, Khaireddine Mrad Dali, Alia Zehani Kassar, Ahmed Sellami, Sami Ben Rhouma, Yassine Nouira

**Affiliations:** Urology Department, La Rabta Hospital, Tunis, Tunisia; Urology Department, La Rabta Hospital, Tunis, Tunisia; Urology Department, La Rabta Hospital, Tunis, Tunisia; Urology Department, La Rabta Hospital, Tunis, Tunisia; Urology Department, La Rabta Hospital, Tunis, Tunisia; Bacteriology Department La Rabta, Tunis, Tunisia; Urology Department, La Rabta Hospital, Tunis, Tunisia; Urology Department, La Rabta Hospital, Tunis, Tunisia; Urology Department, La Rabta Hospital, Tunis, Tunisia

**Keywords:** Xanthogranulomatous, pyelonephritis, cyst, case report

## Abstract

Xanthogranulomatous pyelonephritis may, rarely, occur as a renal cystic mass. We report a case report of a 50-year-old with a history of medically treated renal lithiasis, who consults for left low back pain. Imaging findings concluded to a Bosniak type-3 hemorrhagic cystic mass of the left kidney. The diagnosis of xanthogranumolatous pyelonephritis on its focal form was made histologically. The diagnosis of xanthogranulomatous pyelonephritis is often difficult even with surgical findings and frequently a histological surprise. This points out the importance of identifying it in pre-operative staging; the diagnosis may be suggested by the association of chronic pyelonephritis, renal stones and hypovascular renal tumor syndrome without specificity at sonography and CT.

## INTRODUCTION

Xanthogranulomatous pyelonephritis (XGP) is a rare form of chronic infection of the renal parenchyma which is histologically defined as a combination of chronic pyelonephritis lesions and xanthogranulomatous spume cells. Its pre-operative diagnosis is difficult due to the lack of evidence of specificity [1, 2]. There are two aspects in which PXG presents, as a diffuse one, the most frequent, corresponding in fact to pyonephrosis and another pseudo-tumor focal form posing the problem of differential diagnosis with a renal tumor [1].

We report a case of pseudo tumoral xanthogranulomatous pyelonephritis whose preoperative diagnosis was in favor of a cystic tumor, pointing out the pre-operative diagnostic difficulties of this affection.

## CASE REPORT

**Figure 1:**
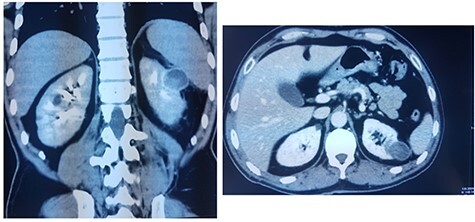
CT showing a heterogeneous Bosniak type-3 cystic mass having a thickened wall.

**Figure 2:**
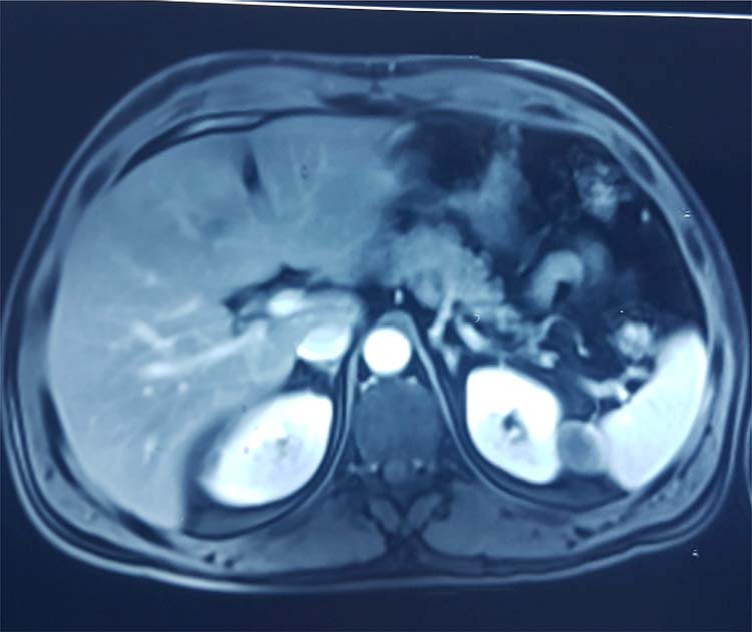
MRI showing left polar cyst having an exophytic development and a thickened wall with spontaneous T2 hypo-intensity and T1 hyperintensity.

**Figure 3:**
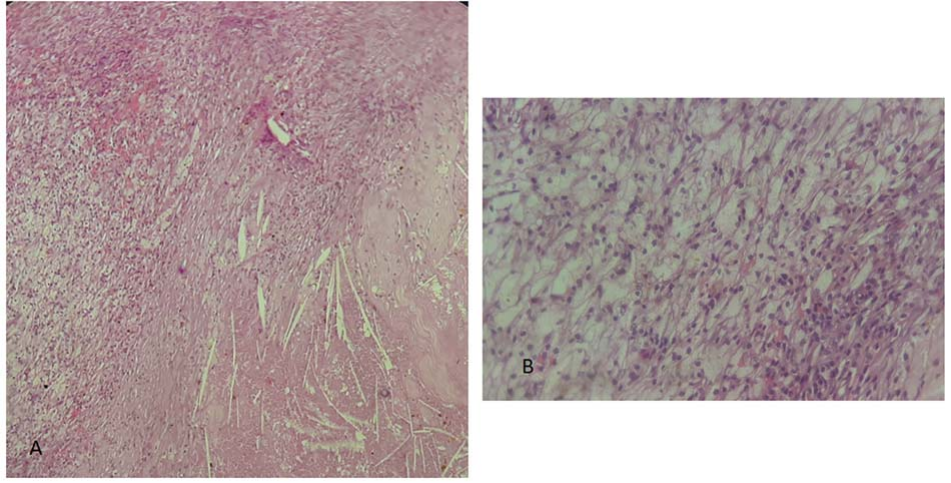
histological examination. (A) Hematoxylin Eosin × 20: inflammatory granulomatous changes in contact with cholesterol crystals. (B) Hematoxylin Eosin × 40: inflammatory infiltrate rich in xanthemous foam cells.

This is a 50-year-old patient with a history of medically treated renal lithiasis, who consults for left low back pain. The patient was apyretic and reported no urinary symptoms. The clinical examination was without any particularities. There was no organomegaly or lumbar contact on bimanual palpation. Biologically, there were no abnormalities, including no biological inflammatory syndrome, and renal function was preserved. The urine culture was sterile. Renal ultrasound revealed a 3-cm left cortical renal mass with moderate vascuarization. Abdominal CT scan confirmed the presence of a heterogeneous Bosniak type 3 cystic mass having a thickened wall (Fig. 1). Abdominal MRI showed a left polar cyst having an exophytic development and a thickened wall with spontaneous T2 hypointensity and T1 hyperintensity without any pathological enhancement after Gadolinium injection (Fig. 2). The retained diagnosis was an hemorrhagic benign cyst. At multidisciplinary meeting, the decision was to perform an MRI 3 months later which showed a left medio-renal cortical cystic mass measuring 24 × 28 mm having a T1 hyperintensity and heterogeneous T2 hypointensity, with a thickened wall mildly enhanced after contrast injection evoking a Bosniak type-3 hemorrhagic cyst. Seeing the MRI results, we decided to perform a left tumorectomy. Pre-operatively, the perirenal tissues were inflammatory and adherent to the capsule, a complete dissection of the kidney was performed allowing the identification of the tumor which was of a 3-cm diameter and a partially exophyctic development, then we cut the tumor with a 2-mm surgical margin. No urinary tract leakage was observed. Finally, renorraphy was performed in two plans using Vicryl 2-0 and 1 (Ethicon, Cincinnati, OH, USA). After confirming the lack of active bleeding, we placed a drainage tube and closed the incision. The total operation time was 1 h 45 min, with 18 min of selective clamping. The post-operative course was uneventful, and the patient was discharged 2 days post-operatively. The histopathological examination revealed a 2.5 × 2.7 cm necrotic lesion with an inflammatory interstitial infiltrate dissociating the epithelial structures (Fig. 3). The retained diagnosis was a pseudo tumoral Xanthogranulomatous pyelonephritis. The CT performed 3-months after surgery showed no abnormalities.

## DISCUSSION

XGP is a rare chronic inflammatory disease firstly described in 1916 [3] whose main cause is due to chronic obstructive urinary tract infections and suppurations of the renal parenchyma. Renal calculi, frequently staghorn stones, may be seen in up to 100% of the published cases [4]. Altered immune response and intrinsic disturbance of leukocyte function have been reported to be possible risk factors [5, 6]. XGP is frequently unilateral and bilateral cases of XGP are extremely rare. Shah *et al.* [7] reported one bilateral XGP child case managed non-surgically.

The physical examination in case of XGP is not of great diagnostic value because it is most often negative. A lumbar mass is only found in 20% of cases [1]. This lack of symptoms leads in most cases to a late diagnosis which is usually made on histological examination. In biology, we may find some non-specific abnormalities such an inflammatory anemia related to the chronic infectious process [4]. The rate of positive urine cultures in case of XGP ranges from 52 to 100% in the literature [1, 3, 4]. The most found bacteria are *Escherichia coli* and *Proteus mirabilis* [1, 8].

Radiologically, the pre-operative diagnosis of XGP is only made in about half of cases from CT data. In the localized form of XGP, CT often reveals a cortical, hypodense and heterogeneous mass with calcifications in some cases. After contrast injection, the mass does not usually increase in density; however, an intense peripheral enhancement corresponding to compressed healthy renal tissue and/or inflammatory tissue is shown [8, 9]. The CT appearance of XGP can mimic clear cell carcinoma, cystic, necrotic or infected kidney tumors, or even some forms of lymphoma [8]. Ichaoui *et al.* [10] reported that a cystic mass of the kidney was suspected in 10% of cases in their series of 42 patients but the histological examination confirmed the diagnosis of PXG. MRI is usually performed as part of a kidney tumor work-up. In fact, MRI seems to establish the diagnosis of focal XGP with better specificity due to its good characterization of adipose tissue [11, 12].

XGP induces a variable and non-specific clinical and radiologic picture. Moreover, there is no pathognomonic clinical or radiologic sign of this condition as it resembles other inflammatory or neoplastic renal pathologies. The diagnosis is usually made on histological examination after surgical treatment.

The histological examination usually shows a dense inflammatory infiltrate of all interstitial tissue with presence of lymphocytes, plasmocyts and neutrophils. We notice a replacement of renal parenchyma with CD68+ foamy histiocytes, occasional multinucleated giant cells and inflammatory cells.

The treatment of focal XGP is both medical and surgical [1, 8]. Although the effectiveness of medical treatment based on antibiotic therapy alone has been reported in some cases of focal XGP like Fitouri *et al.* [13] who reported an XGP case confirmed by percutaneous biopsy. And successfully treated with 8 weeks’ antibiotic therapy. Most series recommend a conservative surgical treatment consisting of partial nephrectomy or limited resection of the lesion after an empiric or adapted antibiotic therapy. In our case, our patient did not receive an antibiotic therapy because the diagnosis XGP in its focal form was never suspected, and urine culture was negative.

## CONCLUSION

XGP in its focal form is a rare benign disease of the kidney. Its treatment should be conservative based on partial nephrectomy or tumorectomy with antibiotic therapy. Lack of knowledge of this disease and the absence of clinic or radiologic specific signs may explain the high rate of misdiagnosis.

## CONFLICT OF INTEREST STATEMENT

The authors declare that they have no competing interests.

## CONSENT

Authors declared that they received written consent from the patient to publish this case report.
